# Growth and dislocation studies of β-HMX

**DOI:** 10.1186/s13065-014-0075-y

**Published:** 2014-12-19

**Authors:** Hugh G Gallagher, John N Sherwood, Ranko M Vrcelj

**Affiliations:** WESTCHEM, Department of Pure and Applied Chemistry, University of Strathclyde, Thomas Graham Building, 295 Cathedral Street, Glasgow, G1 1XL UK; Current address: Chemistry, Faculty of Natural and Environmental Sciences, University of Southampton, Highfield Campus, Southampton, SO17 1BJ UK

**Keywords:** Crystal growth, Dislocations, Etching, Energetics, HMX

## Abstract

**Background:**

The defect structure of organic materials is important as it plays a major role in their crystal growth properties. It also can play a subcritical role in “hot-spot” detonation processes of energetics and one such energetic is cyclotetramethylene-tetranitramine, in the commonly used beta form (β-HMX).

**Results:**

The as-grown crystals grown by evaporation from acetone show prismatic, tabular and columnar habits, all with {011}, {110}, (010) and (101) faces. Etching on (010) surfaces revealed three different types of etch pits, two of which could be identified with either pure screw or pure edge dislocations, the third is shown to be an artifact of the twinning process that this material undergoes. Examination of the {011} and {110} surfaces show only one type of etch pit on each surface; however their natural asymmetry precludes the easy identification of their Burgers vector or dislocation type. Etching of cleaved {011} surfaces demonstrates that the etch pits can be associated with line dislocations. All dislocations appear randomly on the crystal surfaces and do not form alignments characteristic of mechanical deformation by dislocation slip.

**Conclusions:**

Crystals of β-HMX grown from acetone show good morphological agreement with that predicted by modelling, with three distinct crystal habits observed depending upon the supersaturation of the growth solution. Prismatic habit was favoured at low supersaturation, while tabular and columnar crystals were predominant at higher super saturations. The twin plane in β-HMX was identified as a (101) reflection plane. The low plasticity of β-HMX is shown by the lack of etch pit alignments corresponding to mechanically induced dislocation arrays. On untwinned {010} faces, two types of dislocations exist, pure edge dislocations with b = [010] and pure screw dislocations with b = [010]. On twinned (010) faces, a third dislocation type exists and it is proposed that these pits are associated with pure screw dislocations with b = [010].

**Graphical abstract:**
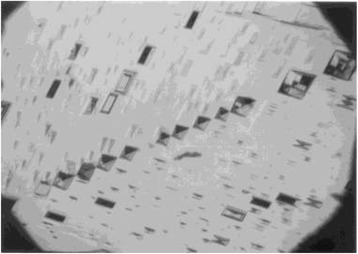
Etch pits on the twinned (010) face of β-HMX.

## Introduction

Understanding the crystal growth, morphology and defect structure of organic materials has become increasingly important as the breadth of their commercial usage increases. Whilst most recently the emphasis has been on pharmaceutical materials, an improved understanding of such properties is required for all forms of crystalline organic materials. An important sub- group is that of the energetic materials A small number of this group have been thoroughly studied, however there is a dearth of information regarding the major energetic material Cyclotetramethylene Tetranitramine (C_4_H_8_N_8_O_8_, HMX). HMX is one of a number of commonly used energetic materials and is a common constituent of plastic bonded explosives; in a similar manner to materials such as RDX and PETN. HMX occurs in four polymorphic forms, α, β, δ and ε [1-6] (and also a hydrated form, termed γ [7]), of these the most stable thermodynamically is β-HMX. This form is monoclinic, space group P2_1_/n, a_0_ = 6.526 Å, b_0_ = 11.037 Å, c_0_ = 7.364 Å, β = 102.66°. The molecules are not hydrogen bonded within the structure (as would be expected from the available functional groups) and so the packing is dominated by van der Waals forces.

The growth of β-HMX crystals has been reported previously by several authors. McCrone [8] obtained massive crystals exhibiting forms {110}, {011}, and {101} by crystallization from acetic acid, acetone, nitric acid and nitromethane. As part of their studies on mechanical deformation of β-HMX, Palmer and Field [9] grew crystals with typical dimensions 5×5×3 mm^3^ by solvent evaporation from acetone solution. They have described single and growth twinned crystals with forms {010}, {011} and {110}. The twinning plane was noted as being a (101) mirror plane. Occurrence of twinning in commercial grade β-HMX has been studied by Kohlbeck and Dubois [10]. They found that twinning was common and that twins followed a normal twin law on (101) planes. None of these reports discussed the perfection of the crystals obtained. As crystal growth is most often driven by defects, a parallel study of the defect structure permits a great deal more information to be developed for any given system. As well as the intrinsic factors that dislocations control, such as crystal growth and mechanical properties, they can play a significant role in such processes as thermal decomposition [11,12]. Dislocation etching is a useful technique for the study of crystal imperfections. It can be used to reveal the points of emergence of dislocations at the surface of an as grown crystal and is particularly useful for examining crystals containing high densities of dislocations. It serves as a useful complement to the more informative technique of Lang X-ray topography, which is limited to crystals with relatively low defect contents. Etching can still offer much information however and has been used on a small range of pharmaceuticals [13-17], proteins [18] and explosives [19,20].

In the case of energetic materials, defect studies have assumed more importance since it was proposed that energy localization due to plastic deformation and dislocation pile-up (“hot spots”) could contribute significantly to the detonation initialization process [21,22]. Whilst dislocation based hot spots are sub-critical in nature, they could contribute to these hot spot processes, especially as crystals grown and mechanically deformed during processing are more than likely to be highly defective in nature. The success of the hot spot model has shown that a good understanding of the defect structure of energetic materials is important in a full characterization of their mechanical properties and to their possible stability [23,24]. As such, a number of studies have been made of these types of materials. The defect structures of RDX and PETN have been thoroughly examined using X-ray topography [25-29], etching, microindentation [19,20] and frictional methods [30] as has that of 2-4-6 Trinitrotoluene (TNT) by X-ray topography [31].

This has not been the case for Cyclotetramethylene-Tetranitramine (HMX), where a limited amount of work has been performed utilizing frictional properties [30] and Palmer and Field’s study with Vickers indentation methods, wettabality and compression techniques, using etching to validate their conclusions [9], but not as a general guide to the dislocation structure of this material. In this paper, solvent growth of β-HMX is described, as are dislocation studies by the etching method.

In the following paper, we use the standard crystallographic bracket notation; parentheses (hkl) for specific Miller planes, square brackets [hkl] for Miller plane direction and braces {hkl} for a family of similarly numbered Miller planes.

## Experimental methods

### Crystal growth and preparation

Standard military grade HMX (purity 99.5%) in wetted powder form was obtained from PERME, Waltham Abbey. The solid was purified before use by multiple crystallisations from distilled acetone. The growth of spontaneously nucleated β-HMX crystals from acetone (Sigma-Aldrich, purity 99%+) by solvent evaporation at 293 K was used mainly for the production of small seed crystals (<0.5 cm^3^), although by slowing the rate of evaporation larger crystals could also be grown by this method.

For seeded crystal growth, previous studies [32] showed a restricted range of solvents that could be used for growing β-HMX, due to its comparatively low solubility in most of the common organic solvents. The published data suggested that the three most suitable solvents were DMSO, butylacetone and acetone (Table 1). Of these, acetone was the least hazardous and consequently the easiest to handle. Additionally, the solubility ratio for β-HMX in acetone, (ds/dT)/s_o_ (K^−1^), where ds/dT is the gradient of the solubility/temperature plot and s_o_ the average solubility in the temperature range examined (381 - 291 K), is 0.026 K^−1^. This figure lies within the range previously defined [33] for the growth of good quality single crystals from solution using standard temperature lowering of a saturated solution method, although the solubility itself is lower than that previously noted as ideal for the production of crystals of high perfection. Using this method, crystals (>1 cm^3^) of HMX were grown with temperature lowering at the rate of 0.05 K hr^−1^ in the range 318 - 291 K.

**Table 1:** **Solubility and solubility ratio of HMX in various solvents**

**Solvent**		**Solubility**	**Solubility ratio (ds/dT)/s**_**o**_ **(K** ^**−1**^ **)**
	**Temp (K)**	**g HMX/100 g solvent**	
DMSO	298	57	0.05
	333	68	
Butylacetone	298	12	0.014
	333	20	
Acetone	298	1.8	0.026
	333	4.2	

### Dislocation etching

Previous reports of dislocation etching of β-HMX used an etchant composed of 3 parts acetone to 2 parts water applied for 30 to 60 seconds. Attempts at etching our crystals under these conditions yielded many poorly defined pits. Therefore, various other mixtures of acetone and water in different proportions were assessed as suitable etchants. It was found that an increase in the ratio of water to acetone in the mixture resulted in longer etching times, which, although desirable for greater control of the process, was unsatisfactory due to the loss of definition. The most sharply defined pits were obtained from pure acetone, but an etching time of 5 seconds at 293 K made accurate control of etching more difficult. Etching times could be increased by lowering the temperature, but in order to avoid problems of uncertainty in temperature and the possibility of thermal shock, 5 seconds at 293 K was generally used followed by a quench in water at the same temperature to arrest the etching process.

Etching studies were carried out on the as grown (010) and twinned (010) faces, on as grown {011} and {110} facets and on faces cleaved parallel to the (011) plane.

## Results

### Crystal growth

**Figure 1:**
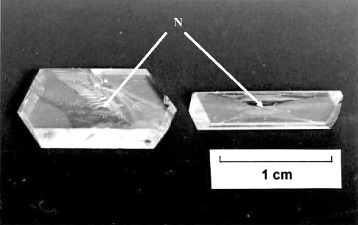
**Typical morphologies for β-HMX crystals grown by slow evaporation from acetone solution.** The nucleation point (N) of each crystal is indicated in the figure.

The crystals obtained by the growth methods employed in this study exhibited only the forms {011}, {110}, (010) and (101) irrespective of growth conditions. The majority of crystals grown by solvent evaporation from spontaneously nucleated solutions had a habit ranging from tabular with {011} dominant to columnar with the elongation along [100]. In addition, a very small number of crystals exhibited a bulky prismatic habit with (101) faces very small and sometimes absent. The morphology of the last were similar to those described by McCrone [8] and by Palmer and Field [9]. Typically the smaller crystals were 6 mm along the longest dimension, although several larger crystals up to a maximum size of 21×10×8 mm^3^ were also obtained. Examples of larger tabular and columnar shaped crystals grown by solvent evaporation are shown in Figure 1.

In contrast, crystals prepared from seeds under more rigorously controlled conditions by slow cooling had only the bulky prismatic habit. The largest of the crystals was 22×14×25 mm^3^. Crystals grown from seeds were generally of poorer quality than those obtained by spontaneous nucleation. Highly defective regions invariably formed in the region of the seed suspension rod and developed towards all growth faces. This damage was always less extensive in crystals grown without stirring. The type of damage that occurred can be seen in the right hand crystal in Figure 1 radiating from the nucleation point in an “upward” direction. This damage was much more prevalent and widely distributed in crystals prepared by slow cooling.

A common feature in the growth of β-HMX was found to be the occurrence of twinned crystals. This was not unexpected. Twinning was observed in all crystals grown from seeds and in at least half of the crystals grown by spontaneous nucleation. Crystals which are normally tabular or columnar retain their basic shape on twinning, but in addition, develop a small “shoulders” at the emergent surface sites of the twins whereas crystals of the prismatic habit which are usually longer along [100] compared with [001] retain a more equant habit. In addition, multiple twinning occurs frequently yielding crystals with complex shapes.

## Results

### General observations

Etching in the manner described produced well-defined etch pits on (010), {011} and {110} growth faces and on the {011} cleavage face. The geometry of the pits depended on the face on which they occurred. Generally all pits were point-bottomed, but some flat-bottomed pits were also observed. Only those pits which remained sharp-bottomed on continued etching could be definitely associated with line defects. The distribution of pits on all surfaces was random and no preferred alignments could be discerned. For all of the crystals studied, the relative pit densities on each face was found to decrease in the order (010) ≈ {011} > {110}.

### Etching of the (010) face

**Figure 2:**
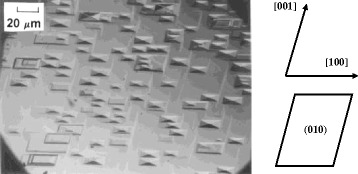
**Optical micrograph of etch pits on the (010) face of β-HMX.**

**Figure 3:**
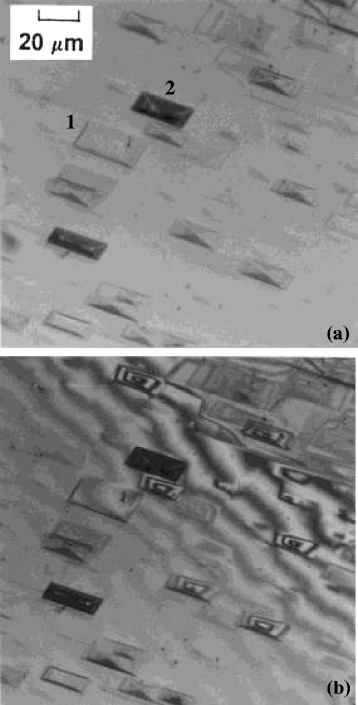
**Etch pits on the (010) face of β-HMX. (a)** Optical and **(b)** interference micrograph, showing type 1 and type 2 etch pits.

Figure 2 shows the general distribution of etch pits on a typical (010) habit face after etching. In detail there are two basic types of pit (Figure 3). Pits of type 1 are smaller in size and appear light in contrast, whereas pits of type 2 are larger and darker. The former type of pit is by far the most predominant. The surface outline of both types of pits are identical, having the shape of a parallelogram with the longer edge aligned along the [100] direction. The measured length to width ratio of almost all the pits had a value of around 2.7:1. In terms of the unit cell, this corresponds to the removal of three unit cells along [100] for every one unit cell in the [001] direction. The three dimensional geometry of the etch pits was revealed using optical and interference microscopy as illustrated in Figure 3. Pits of type 1 exhibit only a few fringes indicating that these are shallow and are bounded by surfaces with high index orientations. In contrast, pits of type 2 show more fringes (thus appear darker) and are therefore much deeper. The small spacing and even distribution of the fringes implies that the sides are steep and planar. The pointed bottom of each pit (type 1 and type 2) coincides with the geometric centre indicating that the associated dislocations emerge normal to the surface. Etch pit densities for different crystals varied in the range 10^5^-10^8^ cm^−2^ for pits of type 1 and 10^2^-10^3^ cm^−2^ for pits of type 2.

### Etching of the twinned {010} face

**Figure 4:**
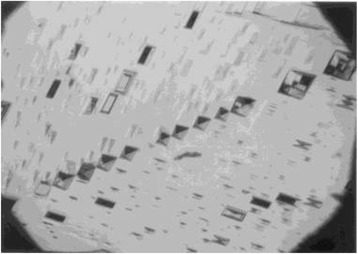
**Optical micrograph of etch pits on the (010) face of growth twinned β-HMX.**

**Figure 5:**
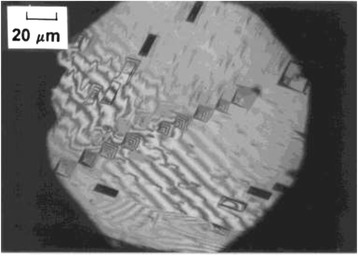
**Interference micrograph of etch pits on the (010) face of growth twinned β-HMX.**

**Figure 6:**
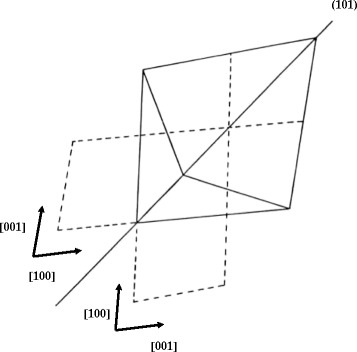
**Reconstruction of an etch pit obtained on the twinned (010) face of β-HMX, showing the (101) twin plane, [100] and [001] directions.**

Typical etch pit patterns on growth twinned {010} facets are shown in Figure 4. Three types of pit geometry are in evidence. Pits of type 1 and type 2 are associated with the untwinned regions of the crystal and are described above. Type 3 pits only occur along the surface trace of the composition plane of the twin. They have a kite-shaped surface outline which shows mirror symmetry associated with twinning about the (101) plane. The edges of the pits are parallel to the [100] and [001] directions. Optical and interference microscopy (Figure 5) indicates that the pits are deep with high index planar sides and the apex lying in the twin plane. This fact, together with pit symmetry, suggests that the dislocation line must lie in the twin boundary. Figure 6 shows how the basic pit geometry can be reconstructed from a type 1 or type 2 pit modified by reflection in the (101) mirror plane of the twin. The additional etching of the tail section that is observed probably arises from the tendency for dissolution to take place at the re-entrant surfaces, caused by the close proximity of the twin, in order to achieve a minimum energy configuration. The dimensions of the pit edges parallel to [100] and [001] directions estimated from many observations expressed as a ratio is 1:1.1. This is in excellent agreement with the relative dimensions of the pits calculated from the model presented in Figure 6. The ratio corresponds to the ratio of the unit cell parameters along the a- and c-axes. It can be concluded that the apex of the pit lies at the centre of the figure and indicates that the dislocation line is normal to the surface. The etch pits are irregularly spaced along the twin plane with typically 10^2^-10^3^ cm^−1^.

### Etching of the {011} face

**Figure 7:**
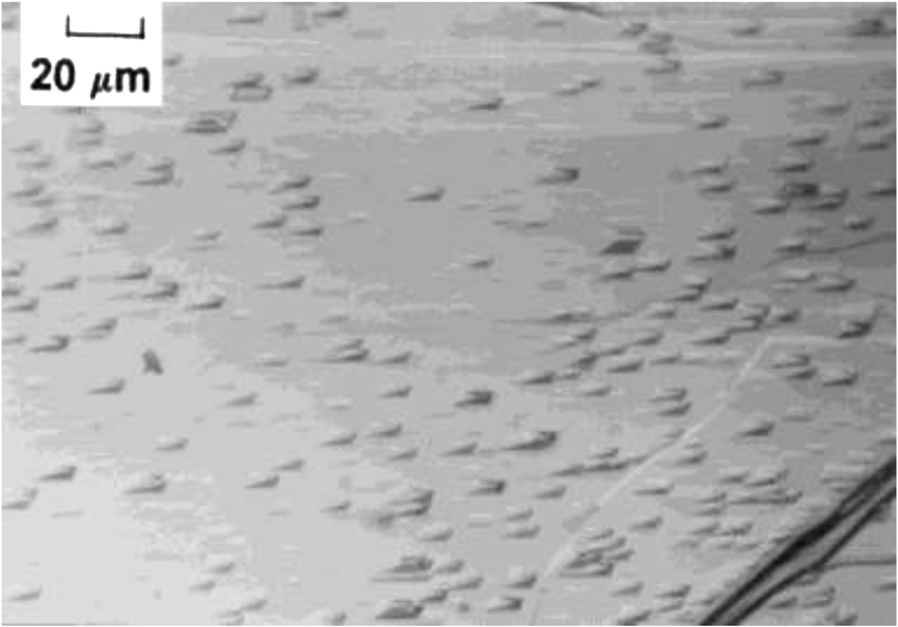
**Optical micrograph of etch pit morphology on the {011} face of β-HMX.**

The outline of the etch pits observed on a {011} face are trapezoidal in shape and are elongated in the [100] direction. An example of such pits is shown in Figure 7. Optical and interference microscopy indicate that each pit is bounded by steep planar surfaces with an apex considerably offset from the centre. This implies that the dislocation line intersects the crystal surface at an oblique angle. Etch pit densities in the range 10^5^-10^8^ cm^−2^ were observed for different crystals.

### Etching of the {110} facet

**Figure 8:**
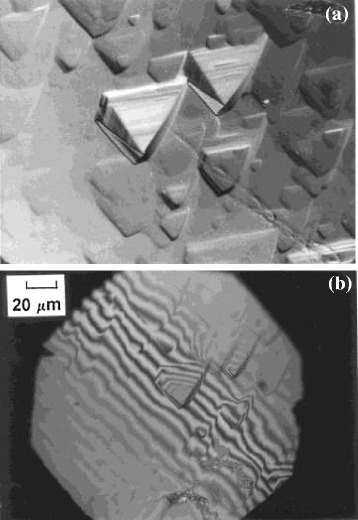
**Etch pit morphology on the {110} face of β-HMX. (a)** Optical and **(b)** interference micrograph.

The etch pits formed on {110} faces are quite different from those observed on the other faces. Only one type of pit, presenting in outline an irregular pentagon is found (Figure 8). Two of the major edges lie along the surfaces traces of {001} planes and a third is parallel to the [001] direction. Interference microscopy shows fringes which indicate that the sides are convex, becoming steeper towards the apex. The shallowness of the pits and an apex which is positioned away from the centre indicate that the dislocations exiting on the {110} faces may be inclined at an angle to the crystal surface. The etch pit density was considerably lower on this face compared to the others and varied in the range 10^3^-10^5^ cm^−2^.

### Correspondence between dislocations and etch pits

**Figure 9:**
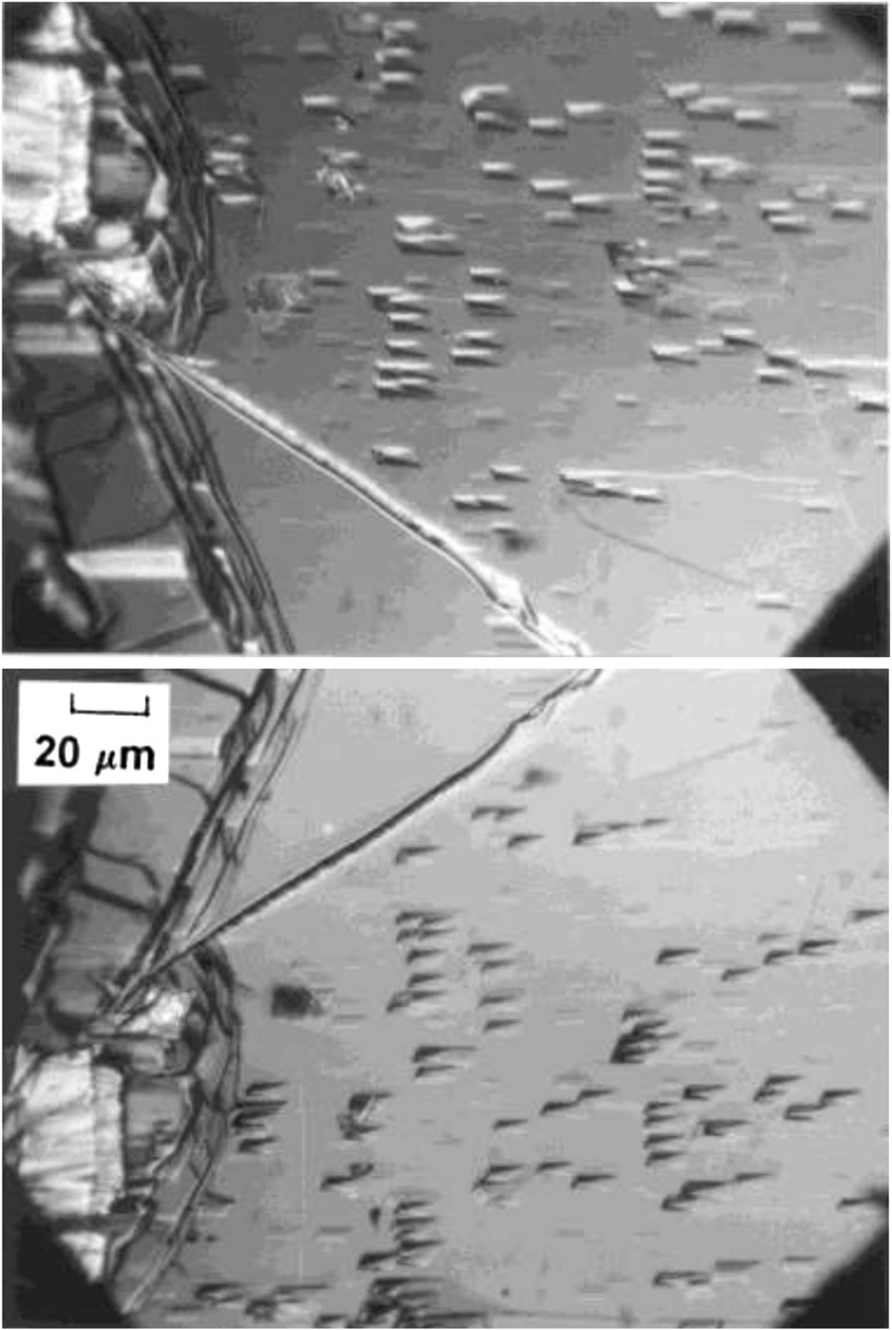
**Matching etch pit patterns on complementary cleavage faces of β-HMX.**

Etch pit patterns can be matched on pairs of cleaved surfaces as seen in Figure 9, confirming that line defects are responsible for etch pits [34,35].

The pits can be followed into the crystal surface on continued etching. They generally remain pointed and retain their shape. Pits that disappear first of all become flat-bottomed suggesting that etchant is sensitive to defects other than dislocations. However, pointed pits appear only at the surface intersection of dislocations. Later studies of localized plastic deformation [36] using a microhardness indenter resulted in an increase in etch pit density around the indentation, with rows of pits aligned along well-defined dislocation slip traces.

## Discussion

**Figure 10:**
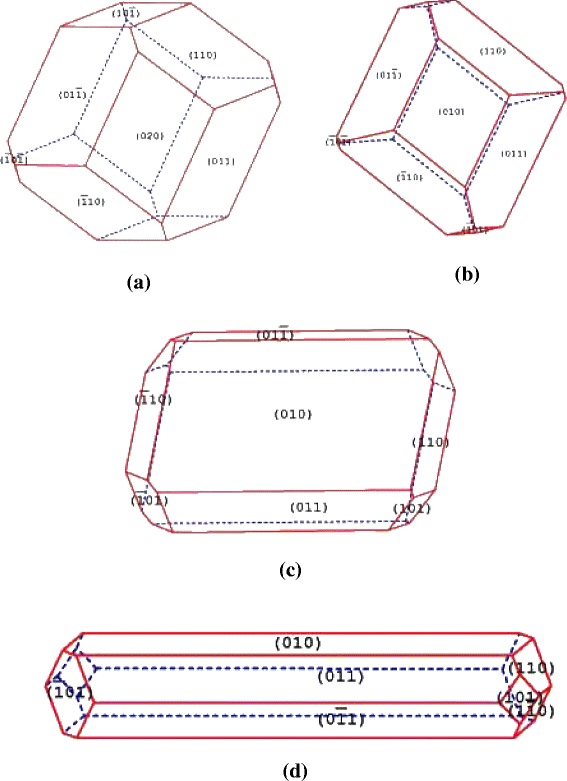
**SHAPE calculations of the equilibrium form of HMX and the expected changes of gradually increasing the relative growth rates of the crystal faces growing in the {100} direction. (a)** Predicted Donnay-Harker morphology of HMX; **(b)** prismatic; **(c)** tabular; **(d)** columnar.

Despite the variations in growth conditions all the crystals of β-HMX grown in this study exhibited the morphological forms {011}, {110}, (010) and (101) only. The law of Donnay and Harker [37] predicts that the predominant crystal forms have the smallest reticular areas and are associated with the largest interplanar spacings. This relatively simple approach is consistent with and explains many aspects of the observed morphology. The calculated reticular areas and interplanar spacings [38] are listed in Table 2. It can be seen that the four smallest reticular areas in order of decreasing morphological importance are {011}, {110}, (010) and (101), in agreement with the observed faces and estimated relative facial areas for typical crystals. The absence of further faces is explained by the considerably larger reticular areas for forms other than those observed. Calculation of the equilibrium form using the prediction programme SHAPE [39] using the Donnay-Harker approach yields the often observed equant prismatic form shown in Figure 10. In the present case however the crystal habit varied significantly with the method of growth.

**Table 2:** **Reticular areas and interplanar spacings for β-HMX**

**Morphological importance**	**Form (hkl)**	**Reticular area**	**Interplanar spacing (Å)**
1	(011)	0.8059	6.0213
2	(110)	0.7691	5.5258
3	(010)	0.7693	5.5250
4	$\left(10\overline{1}\right)$	0.7881	5.3928
5	$\left(11\overline{1}\right)$	0.8720	4.8464
6	(021)	0.9706	4.3789
7	(101)	0.9832	4.3227

The largest variations were observed for crystals grown by self-nucleation on solvent evaporation in unstirred solution. Since crystals of all three habits (prismatic, tabular and columnar) frequently occurred in the same crystallizing dish, it is unlikely that external effects such as impurities or growth temperature alone were responsible for the variations. Such differences in crystal habit are often attributed to localised variations in supersaturation which can readily occur during crystallization from stagnant solution and there is no doubt that these factors complicate the situation, It is more likely however that the situation results from the expected gradual decrease in supersaturation during the growth period. The excessively high supersaturation required to initiate nucleation will decrease, initially rapidly and then more gradually, to lower values as the crystallisation proceeds. This change is accompanied by significant and varied reductions in growth rates of the crystal faces and hence the crystal shape. The fact that growth by solvent evaporation in dishes at higher supersaturation yielded crystals of tabular and columnar habits and the eventual observation as time progressed of a preponderance of prismatic crystals in the growth solution fits well with this suggestion.

Taking the situation in reverse, at the later times of growth much of the solute content of the solution has been expended and the crystals then formed are growing under low supersaturation conditions. That is to say nearer to an equilibrium state which is effectively the situation defined by the Donnay and Harker theory. Hence they show predominantly this predicted equant, prismatic morphology. With increasing supersaturation and potentially for reasons of mechanistic changes in the growth process, the faces propagating in the general <100 > directions grow increasingly more rapidly than the lateral faces to yield successively a tabular and columnar morphology. Returning to the operating direction it is then not surprising that the full range of morphologies are observed with the evaporation technique and in the order of appearance observed, columnar and tabular crystals at the higher initial supersaturations, followed at longer times by prismatic crystals at the lower supersaturations.

In contrast, only minor variations in the prismatic habit were obtained for crystals grown in crystallizers. This consistency is attributable to the more rigorously defined and controlled conditions of supersaturation imposed during growth by this method. A seed is used to initiate growth and the significant effects of nucleation are eliminated. The growth rates of the faces are held in a more constant low supersaturation growth rate regime which happens to be that which yields only prismatic morphologies. This would appear to be the ideal situation but, as noted above, the supersaturation conditions defined by the properties of the solvent were perhaps not ideal and it is possibly this factor, coupled with the unusual mechanical behaviour of HMX that led to crystals of lower quality using this technique. Future improvements in quality can only come from an evaluation of the influence of the supersaturation dependence of the growth mechanism of the material and hence a better definition of the conditions for better growth.

**Figure 11:**
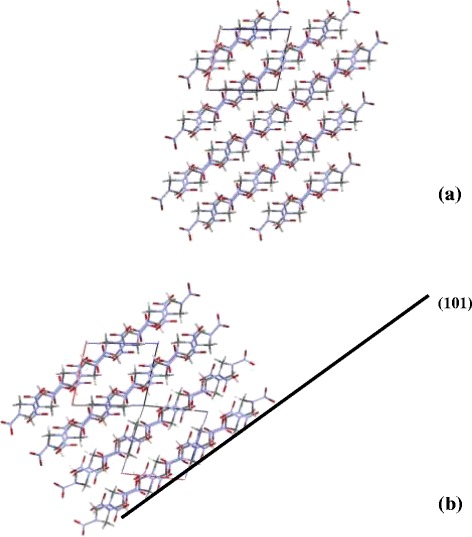
**Unit cell of β-HMX projected on the (010) face (a) normal structure (b) twinned structure.**

Growth twinning was found to occur by reflection in (101) planes, in agreement with previous observations [9]. The frequency of occurrence suggests that twinning is an energetically inexpensive process. Figure 11a shows the unit cell of β-HMX projected on the (010) plane. The HMX molecule is centrosymmetric and exists in a slightly puckered chair conformation. The crystallographic structure consists of layers of these molecules stacked parallel to the (101) plane. Apart from the nitramine groups which project outwards, above and below the plane of the ring, the structure is reasonably open and can accommodate the minor deviations in packing involved in twinning. The twinned structure viewed along [010] is shown in Figure 11b.

An important contribution to the packing energy is derived from the bonding interaction between the oxygen atoms of the nitramine groups on one molecule and the adjacent ring carbons on neighbouring molecules. Since twinning occurs readily, the small distortions due to the new positions of the nitramine groups at the twin boundary must be considered to have little effect on bonding energies. β-HMX is brittle and tends to fracture and twin under a fairly low mechanical stress. The lower quality of the crystals grown from seeds is most likely the result of mechanical deformation during seed preparation and crystal growth.

Drilling the hole required for the seed suspension fibre introduces small microcracks or microtwins which can propagate into the crystal during growth or act as the sites for nucleation of larger defects under the stresses produced by rotation of the crystal while stirring the solution. This appears consistent with the observation that the extent of damage and twinning is considerably reduced, although not altogether removed, when stirring is not employed.

Evidence accumulated from etching (and as shown in a subsequent paper, describing microindentation studies) strongly suggests that the observed etch pits are associated with emergence of dislocations. Furthermore, it is believed that the dislocations responsible for the pits correspond to growth dislocations only and not mechanically induced dislocations. This view is supported by the following observations: the pits persist into the crystal during successive etches, they are randomly distributed and do not form part of any alignments. Mechanically induced dislocations on the other hand are expected to form arrays along preferred directions, in which pairs of pits rapidly develop flat bottoms and disappear on continued etching. Dislocations of this type, which generally result from surface damage during growth and subsequent handling, have previously been observed in PETN [19] grown in the same way. Absence of dislocations with a mechanical origin is indicative of the low plasticity of β-HMX.

The etch pits formed with characteristic shape and size depending on the molecular packing at the face and the nature of the dislocation itself. The influence of crystal packing is clearly demonstrated by several aspects of etch pit geometry. On (010) faces, the relative dimensions of the pit edges are related to an integral ratio of unit cell dimensions for the three types of pit observed. The shape of pits on each of the habit faces is different, although in each case the pits are bounded by planes which intersect the surface along well-defined and often low index crystallographic directions. These variations in etch pit shape reflect the symmetry of the underlying crystal structure. β-HMX crystallizes in the space group P2_1_/n which is a subset of the Laué class point group 2/m. Clearly there is a two-fold rotational symmetry along [010] and subsequently no obvious basic symmetry on {011} and {110}. This predicted symmetry is consistent with the symmetry of the observed etch pit shapes. Pits occurring on (010) are regular quadrilaterals having two-fold rotational symmetry, while those on {011} and {110} are asymmetric. Further evidence of the relationship between molecular packing and etch pit shape is found on the twinned (010) surface and as an additional bonus tends to confirm (101) as the twin plane. Twinning by reflection in (101) planes result in mirror symmetry about these planes which is reflected in the symmetry of the kite-shaped etch pits in surface section lying along the twin boundary.

Preferential dissolution at dislocation sites is a consequence of the strain energy associated with the dislocation core. Dislocations of different character have dissimilar strain fields and may give rise to variations in etch pit geometry. Halfpenny et al. [19] were able to identify dislocations of different character on {110} faces of PETN crystals from differences in observed etch pit depth. Screw dislocations in general etch at a faster rate than edge dislocations and are therefore larger in size and deeper. Conversely, all of the pits on a surface having identical shape must correspond to dislocations of the same character.

On the (010) faces, all of the dislocations emerge normal to the surface and therefore probably correspond to pure edge or pure screw dislocations. The greater size and depth of the type 2 pits suggests that they are formed at the emergent ends of pure screw dislocations if one applies the above criterion, and that the smaller, shallower type 1 pits result from pure edge dislocations. The five most likely Burgers vectors are [100], [001], [101], $\left[10\overline{1}\right]$ and [010] (∣b∣ = 0.654 nm, 0.736 nm, 0.873 nm, 1.084 nm and 1.105 nm respectively). Of these only the high energy b = [010] corresponds to a pure screw dislocation. On present evidence therefore type two pits are associated with pure screw dislocations with b = [010]. The remaining Burgers vectors listed are all perpendicular to [010] and are therefore pure edge in character. On energetic grounds it is likely that pits of type 1 result from pure edge dislocations with b = [100].

Line directions of dislocations lying along the twin boundary are normal to the (010) surface. As the Burgers vector for a dislocation lying in a twin plane must also be contained in this plane, the possible dislocations are restricted to those with b = [010] or b = $\left[10\overline{1}\right]$. Since etch pits associated with these dislocations have the same basic shape as those obtained in untwinned regions, it seems likely that dislocations have the same character as those found elsewhere on the face. It is proposed therefore that type 3 etch pits originate from pure screw dislocations with b = [010] situated at the twin boundary.

Although type 3 etch pits are aligned due to their association with a twin boundary, they are randomly positioned and the number of etch pits per cm^2^ is statistically identical to that obtained by counting type 2 pits along any imaginary line parallel to the surface trace of the (101) plane. This strongly suggests that these dislocations associated with the twin plane are normal growth dislocations and play no part in accommodating strains arising at the boundary due to twinning.

Although a combination of optical and interference microscope techniques can sometimes be used to determine the line direction of dislocations, their use is limited to such cases in which the etch pits display favourable symmetry. In the case of the {011} and {110} faces, their inherent asymmetry prohibits any deductions regarding the nature of emergent dislocations. However, the fact that only one type of pit is observed on each face implies that all dislocations intersecting that particular face are of the same character.

Further studies using X-ray topography were not possible as the β-HMX crystals had dislocation densities at the limit or exceeded that suitable for such imaging methods and so etching was considered to be the only satisfactory method for the analysis in this instance.

## Conclusions

Crystals of β-HMX were grown from acetone solution by solvent evaporation of spontaneously nucleated solutions and by solvent evaporation and slow cooling of seeded solutions. The crystal forms exhibited were {011}, {110}, (010) and (101) irrespective of growth conditions. This is in agreement with the morphology predicted from reticular area calculations which also explain the absence of additional forms. Three distinct crystal habits were observed, depending on the supersaturation of the growth solution. Crystals of the prismatic habit were favoured by growth under conditions of low supersaturation, while tabular and columnar crystals were predominant at higher super saturations. The observation of crystals of all three habits during growth by solvent evaporation in unstirred solutions was attributed to localised and general variations in supersaturation. Many crystals were found to be twinned. The twin plane was identified as a (101) reflection plane, confirming previous observations by other workers. Crystals grown by spontaneous nucleation were generally of greater perfection than those obtained from seeds. This appears a consequence of mechanical damage introduced during seed preparation and crystal growth.

For these crystals of β-HMX, the dislocation density was so high that for dislocation studies, X-ray topography could not be used and only etching studies would suitably define the dislocations associated with the as-grown crystals, although a more careful selection of growth conditions may yet yield crystals with suitable defect densities for topographic imaging. Although our etching conditions vary markedly with those of previous authors, our prerequisites for the final state of the etch pits was different from those of Palmer and Field, so we ascribe these differences to a simple difference in methodology to achieve different outcomes. The correspondence between etch pits and dislocations is clear – in particular, the evidence of the continuation of etch pits across a cleaved crystal surface show that these are not simply due to mechanical damage during processing. In addition to this, the random spread of etch pits and absence of etch pit alignments corresponding to mechanically induced dislocation arrays was considered to reflect the low plasticity of β-HMX. On untwinned {010} faces, two types of dislocations emerging normal to the face were identified from differences in size and depth of the etch pits. Dislocations associated with pits of type 1 were assigned as pure edge dislocations with b = [010] and those associated with type 2 pits as pure screw dislocations with b = [010]. On twinned (010) faces, pits which lie in the twin plane (type 3 etch pits) correspond to growth dislocations which also lie normal to the surface. It is proposed that these pits are associated with pure screw dislocations with b = [010]. The difference in shape of type 3 pits compared with type 2 pits is a consequence of crystal packing along the twin boundary; however these dislocations are not implicated in the accommodation of stresses due to twinning. The nature of the dislocations intersecting {011} and {110} surfaces could not be determined using etching techniques due to the asymmetry associated with the etch pits. A final definition of these remaining dislocation types will require a more detailed examination of the etch pit structure or the growth of well-defined crystals and their examination by X-ray topographic methods. Nevertheless, etching studies have revealed the dislocation structure on the major face of β-HMX and yield basic evidence as to its low plasticity.
